# From Extraction to Cryobanking: Which Critical Process Parameters Genuinely Improve DPSC Production?

**DOI:** 10.3390/ph19030429

**Published:** 2026-03-07

**Authors:** Tomasz Gedrange, Benita Wiatrak, Tomasz Gębarowski, Ewa Barg, Łucja Cwynar-Zając, Katarzyna Gębczak, Helena Moreira, Aneta Cieśla-Niechwiadowicz, Jakub Hadzik, Amelie Lupp, Sophie Gedrange, Marzena Dominiak

**Affiliations:** 1 Department of Dental Surgery, Wroclaw Medical University, Krakowska 26, 50-425 Wroclaw, Poland; tomasz.gedrange@umw.edu.pl (T.G.); jakub.hadzik@umw.edu.pl (J.H.); marzena.dominiak@umw.edu.pl (M.D.); 2 Institute of Pharmacology and Toxicology, Jena University Hospital, Friedrich Schiller University Jena, Drackendorfer Straße 1, D-07747 Jena, Germany; amelie.lupp@med.uni-jena.de; 3 Department of Pharmacology, Faculty of Medicine, Wroclaw Medical University, Mikulicza-Radeckiego 2, 50-345 Wroclaw, Poland; 4 Department of Biostructure and Animal Physiology, Wroclaw University of Environmental and Life Sciences, Kożuchowska 1/3, 51-631 Wroclaw, Poland; tomasz.gebarowski@upwr.edu.pl (T.G.); aneta.ciesla-niechwiadowicz@upwr.edu.pl (A.C.-N.); 5 Department of Basic Medical Sciences and Immunology, Faculty of Pharmacy, Wroclaw Medical University, Borowska 211, 50-556 Wroclaw, Poland; ewa.barg@umw.edu.pl (E.B.); lucja.cwynar-zajac@umw.edu.pl (Ł.C.-Z.); katarzyna.gebczak@umw.edu.pl (K.G.); helena.moreira@umw.edu.pl (H.M.); 6 Student Research Group, Department of Dental Surgery, Faculty of Dentistry, Wroclaw Medical University, Krakowska 26, 50-425 Wroclaw, Poland; sophie.gedrange@umw.edu.pl

**Keywords:** ATMP, dental pulp-derived cells, DPSCs, vitamin D, 25(OH)D, collagenase II, passages, cell yield, CQAs, BH-FDR, HC3, sensitivity analysis

## Abstract

**Background**: Advanced therapy medicinal products (ATMPs) require strict control of critical process parameters (CPPs) to ensure manufacturing efficiency. The relative impact of donor systemic factors, such as vitamin D status, versus technical process parameters on dental pulp-derived stem cell (DPSC) production remains unclear. **Methods**: In this prospective observational study, 250 adults undergoing extraction of impacted mandibular third molars were included. Dental pulp was processed under a standardized SOP using different preparation methods and enzyme conditions. Primary endpoints were serum 25(OH)D concentration and cell yield; secondary endpoints included number of passages and cryovials. **Results**: Mean 25(OH)D concentration was 30.1 ± 14.5 ng/mL and was higher in supplemented individuals (38.2 ± 14.0 vs. 25.6 ± 12.7 ng/mL; *p* < 0.0001) but was not associated with cell yield (ρ = 0.14, *p* = 0.168) or passages (ρ = 0.07, *p* = 0.406). In contrast, process parameters showed strong effects: scissor preparation resulted in a substantially higher yield than mechanical methods (median 5.00 vs. 1.00 million cells; *p* = 3.6 × 10^−13^), and type II collagenase was independently associated with a higher yield (+2.04 million cells; *p* = 0.026). The number of passages was the strongest predictor of yield (β = 2.28 million per passage; *p* < 10^−26^). Post-thaw viability remained high (mean 90.1% and range 81–98%). **Conclusions**: Manufacturing efficiency of DPSCs is primarily determined by critical process parameters, particularly preparation method, enzyme selection, and passage control, whereas donor vitamin D status did not significantly influence outcomes under the studied SOP. These findings highlight process standardization as the key driver of reproducible ATMP manufacturing.

## 1. Introduction

Dental pulp stem cells are multipotent and can differentiate into various cell types, such as bone, nerve, and fat cells. Wisdom teeth are considered to be a particularly rich source of these cells. Compared to other areas, cell harvesting is associated with less pain. These cells exhibit strong proliferative capacity and multipotent differentiation potential, making them highly suitable for regenerative medicine and translational research applications.

Advanced therapy medicinal products (ATMPs)—including gene, cell, and tissue-engineered products—are regulated in the European Union by Regulation (EC) No 1394/2007 and the associated EMA guidelines, which emphasize defining and verifying critical quality attributes (CQAs), including identity, purity, potency, and stability [1,2,3].

One of the key bottlenecks in ATMP implementation is the availability of suitable cellular material. Autologous models minimize immunological risk but are burdened by donor variability, prolonged timelines, and high manufacturing costs. An alternative is the development of allogeneic, off-the-shelf products that shorten time to access but require engineering to reduce immunogenicity and must meet additional requirements for quality comparability and pharmacokinetics [4,5].

Regardless of the manufacturing strategy, rigorous cell quality assessment and validation of potency assays linked to the mechanism of action remain crucial for safety and efficacy [2,3]. In practice, this entails simultaneously accounting for technical process factors (isolation method, culture medium, number of passages, and cell yield) and biological donor/patient characteristics.

Among modifiable biological factors, the vitamin D axis is noteworthy. That is to say, 25-hydroxyvitamin D (25(OH)D) reflects vitamin D status and exerts broad immunomodulatory effects on T and B lymphocytes and innate immune cells [6]. Preclinical in vitro and ex vivo studies suggest that vitamin D3 may enhance the proliferation of human mesenchymal stromal and bone marrow-derived stromal cells (MSCs), support the expression of pluripotency markers and osteogenesis, and modulate their biological functions [7,8]; however, clinical evidence regarding its effect on stem cell manufacturing efficiency remains limited. Importantly, vitamin D signaling has also been shown to influence stem cell production itself. It is demonstrated that vitamin D regulates hematopoietic stem cell (HSC) formation and expansion, indicating that vitamin D may affect not only differentiation but also stem cell yield and regenerative potential [9].

**Figure 1:**
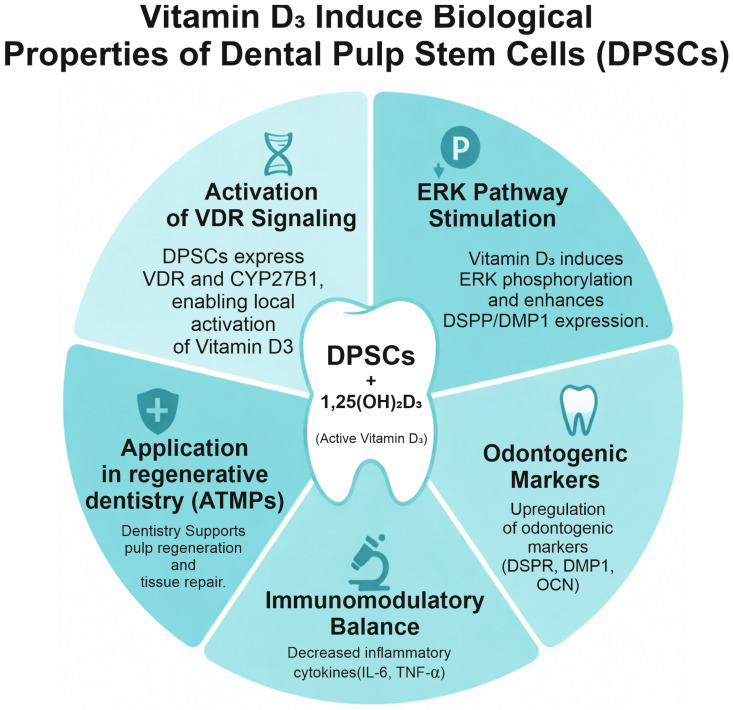
Conceptual map of vitamin D_3_ actions relevant to dental pulp stem cells (DPSCs): vitamin D receptor (VDR) activation, extracellular signal-regulated kinase (ERK) pathway stimulation, up-regulation of odontogenic/osteogenic markers such as dentin sialophosphoprotein (DSPP), dentin matrix protein-1 (DMP1) and osteocalcin (OCN), enhanced alkaline phosphatase (ALP) activity and mineralization, immunomodulatory balance, and potential applications in advanced therapy medicinal products (ATMPs).

Beyond vitamin D, the influences of hormone–metabolic axes are also considered, including growth hormone (GH), which can modulate MSC fate and the osteo-adipogenic balance, with implications for tissue regeneration and potentially for culture efficiency [10]. The proposed mechanisms by which the vitamin D axis influences DPSCs are summarized in Figure 1.

Current expert recommendations in Poland and internationally underline the high prevalence of vitamin D deficiency/insufficiency, with a deficiency threshold around ≤20 ng/mL and target levels of ≥30 ng/mL, although recommended target ranges and supplementation criteria vary across scientific societies [11,12,13].

In the present study, the following were measured: 25(OH)D, cholecalciferol (D3) supplementation, growth hormone (GH), complete blood count parameters, and culture process variables (method, medium, number of passages, cell yield, and number of cryovials). The aim is to situate these observations within the current literature on the availability and quality of cells for ATMP transplantation and to assess the extent to which modifiable nutritional–hormonal factors (including 25(OH)D status and D3 supplementation) may support the standardization of input material in autologous models or indirectly improve biological readiness within the concept of allogeneic off-the-shelf products. However, the relative contribution of donor-related systemic factors, such as vitamin D status and supplementation, versus critical process parameters in determining DPSC manufacturing efficiency under standardized SOP conditions remains insufficiently defined. Therefore, we aimed to determine whether modifiable donor-related factors, including vitamin D status and supplementation or technical process parameters, have a greater impact on DPSC manufacturing outcomes, particularly cell yield and expansion efficiency, under standardized manufacturing conditions.

Beyond vitamin D and growth hormones, important components of the bone mineral axis that influence cellular potential include alkaline phosphatase (ALP), calcium (Ca), inorganic phosphate (P), parathyroid hormone (PTH, intact), and cortisol. ALP is a classical marker of osteoblast activity and early matrix mineralization; Ca/P parameters and PTH concentration are closely linked to interpreting 25(OH)D status and to reflecting compensatory parathyroid activation in vitamin D deficiency. Cortisol, in turn, exerts anti-proliferative and anti-osteogenic effects on stromal cells, potentially modifying cell yield and phenotype in culture. For these reasons, incorporating ALP, Ca, P, PTH, and cortisol into donor/patient evaluation is justified in the context of standardizing the quality of the starting material for ATMPs [11,12,13,14,15].

## 2. Results

### 2.1. Patient Characteristics

A total of 250 consecutive participants with complete 25(OH)D measurements were included (Table 1) and stratified by vitamin D3 supplementation status into a no-supplementation group (*n* = 161) and a D3-supplementation group (*n* = 89); median age was 24.0 [IQR 5.0] vs. 27.0 [IQR 10.0] years, and the females accounted for 110/161 (68.3%) vs. 74/89 (83.1%), respectively. The mean (±SD) 25(OH)D concentration in the entire cohort was 30.1 ± 14.5 ng/mL (median 27.7 ng/mL, IQR 17.3 ng/mL, and range 5.3–84.0 ng/mL). Individuals supplementing with D3 had significantly higher 25(OH)D than those not supplementing (38.2 ± 14.0 vs. 25.6 ± 12.7 ng/mL; Welch’s *t*-test, *p* < 0.0001; Cohen’s d ≈ 0.96). The proportion achieving ≥30 ng/mL was also higher in the supplementation group (52.6% vs. 22.3%; chi-square test, *p* < 0.0001; and Cramér’s V ≈ 0.31).

Other demographic and biochemical characteristics were comparable between groups. There were no significant differences in age (test selected according to distribution) or sex distribution (chi-square/Fisher’s exact test). Markers of the calcium–phosphate metabolism—parathyroid hormone (PTH), total calcium (Ca), and inorganic phosphate (P)—did not differ significantly between groups (all *p* > 0.05). Alkaline phosphatase (ALP) activity, cortisol and growth hormone (GH) levels, and basic complete blood count indices (white blood cells (WBC), red blood cells (RBC), and hemoglobin (Hb)) likewise showed no significant deviations (all *p* > 0.05, effect sizes small, with Cohen’s d or rank-biserial r near zero).

### 2.2. Cell Yield and Passage Number vs. Vitamin D Status and D3 Supplementation

In bivariate analyses, vitamin D status did not relate to the process metrics: 25(OH)D showed no significant correlation with yield (Spearman ρ = 0.14 and p(FDR) = 0.168) or with number of passages (ρ = 0.07 and p(FDR) = 0.406), as summarized in Table 2. By contrast, the passages ↔ yield relationship was strong and linear (Table 2): Pearson r = 0.69 (*p* < 10^−26^), with a concordant Spearman ρ = 0.82 (*p* < 10^−44^).

In multivariable OLS with HC3 robust errors (Table 2), passages remained the dominant independent predictor of yield (β = 2.28 million per passage, 95% CI 1.86–2.69, *p* < 10^−26^, model R^2^ ≈ 0.61). However, 25(OH)D was not an independent predictor of yield (β = 0.02 million per 1 ng/mL, 95% CI −0.01–0.05, *p* = 0.30). In a separate model with 25(OH)D as the outcome, D3 supplementation was associated with a +13.2 ng/mL higher 25(OH)D (95% CI 7.4–19.0; *p* = 8.1 × 10^−6^; R^2^ ≈ 0.29). Taken together—and as consolidated in Table 2—manufacturing efficiency in this SOP is driven primarily by process factors (passages) rather than by systemic markers. While D3 supplementation increased circulating 25(OH)D as expected, neither 25(OH)D concentration nor supplementation status was significantly associated with cell yield, number of passages, or number of cryovials (all *p* > 0.05).

### 2.3. Effect of Preparation Method and Enzyme/Medium on Process Efficiency (And Dependence on D3 Supplementation)

Scissor preparation produced markedly better outcomes: median cell yield was 5.00 million cells [IQR 3.00] with scissor cutting compared with 1.00 million cells [IQR 0.50] using mechanical tearing/pipetting (*p* = 3.6 × 10^−13^; Table 3), representing an approximately five-fold increase in yield. The number of passages and cryovials was likewise significantly higher with scissor preparation. The enzyme/medium also mattered, with type II collagenase achieving the highest values across outcomes; the groups differed significantly on Kruskal–Wallis, and BH-FDR-adjusted post hoc tests retained significance for multiple pairs, with the largest contrasts favoring type II collagenase over dispase/none (Table 4). In OLS-HC3 that was adjusted for systemic markers and demographics, type II collagenase remained independently associated with a higher yield (β = +2.04 million, 95% CI 0.24–3.83, *p* = 0.026), while interactions with D3 supplementation were not significant (Table 4).

### 2.4. Post-Thaw Cell Culture Evaluation

In the whole cohort, post-thaw viability averaged 90.1% (±3.6), with a median of 90% (IQR 4%; range 81–98%) (Table 1). This high post-thaw viability confirms the robustness of the applied cryopreservation protocol and supports its suitability for downstream clinical applications and ATMP manufacturing. The time to the first passage was 9.2 ± 2.7 days, median 9 days (IQR 4; range 4–16). Vitamin D3 supplementation (recorded as a binary yes/no variable due to inconsistent documentation of dose and duration) was not associated with either metric, and the observed effects were small (all *p* > 0.05). A higher post-thaw viability went with a higher yield (Spearman ρ 0.37, p(FDR) 0.044) and with a shorter time to the first passage (ρ −0.58, p(FDR) 0.002). A longer time to the first passage meant a lower yield (ρ −0.43, p(FDR) 0.023) and tended to occur alongside more passages (ρ 0.41, p(FDR) 0.031).

### 2.5. Sensitivity Analysis

After log10 transformation, results remained stable: in the model without passages (Model A), R^2^ = 0.518 (adjusted 0.408) and categories corresponding to type II collagenase retained positive and significant coefficients on the log10 scale; in the model including number of passages (Model B), the model fit increased to R^2^ = 0.798 (adjusted 0.748), with the advantage of type II collagenase still evident, whereas the “scissor cutting” effect attenuated after accounting for the strong influence of passages. The conclusions did not change relative to the main analysis.

D3 supplementation was not associated with the process parameters, despite being associated with higher 25(OH)D concentrations. Manufacturing efficiency was driven primarily by process factors, especially the number of passages, as well as the optimization of tissue preparation and enzymatic digestion, with the combination of scissor cutting and type II collagenase producing the highest yields.

## 3. Discussion

In a uniformly collected cohort of patients undergoing planned extraction of impacted third molars, we demonstrated that the efficiency of producing cells from dental pulp/extracted teeth is determined primarily by process factors, rather than by systemic markers. Specifically: (i) the number of passages was most strongly associated with yield; (ii) the preparation method (“scissor cutting”) and type II collagenase were associated with significantly higher yield and better process metrics; and (iii) vitamin D status—although significantly higher in individuals supplementing D3—did not translate into yield, number of passages, or number of cryovials in the protocol studied. The observed lack of interaction with D3 supplementation suggests that the benefits of optimizing the preparation procedure are independent of the 25(OH)D status [16].

Preclinical data indicate that vitamin D signaling (primarily via 1,25(OH)_2_D/calcitriol acting through the vitamin D receptor, VDR) can modulate the proliferation, adhesion, and osteogenic differentiation of dental pulp cells and other mesenchymal populations [16,17,18,19]. However, the transfer of these effects ex vivo to process outcomes is often constrained by medium composition, enzymatic conditions, and the mechanics of preparation. Classical protocols for isolating DPSCs have used a collagenase/dispase combination, as confirmed by the work of Gronthos and colleagues [19,20]; our findings suggest that, in manufacturing practice, type II collagenase (in combination with scissor cutting) may yield higher efficiency than dispase-containing variants, underscoring the primary role of CPPs (critical process parameters) in ATMP. In this context, the observation that D3 supplementation improves a status marker (25(OH)D) but not process metrics aligns with reports that stated that process standardization had a greater impact on cell yield and “fitness” than donor systemic variability [1,2,3,4,16,17,18,19,20,21,22,23,24,25,26].

In our cohort, D3 intake predominantly fell within 2000–4000 IU/day—doses that improved 25(OH)D yet did not alter manufacturing endpoints. Clinically, these intakes were within or close to commonly cited upper safe ranges, whereas chronically high intakes are far above this (e.g., 10,000 IU/day), exceeding established limits and, so far, raising safety concerns (hypercalcemia, nephrocalcinosis) [11,12,13].

Based on research by Veugelers, P. and Ekwaru, J.P., it was stated that the Institute of Medicine (IOM) made a critical statistical error in deriving the recommended daily allowance (RDA) for vitamin D, misinterpreting the predictive limit for mean study results as applicable to individuals. The current RDA is insufficient for most: The current RDA of 600 IU/day of vitamin D is likely insufficient in ensuring that 97.5% of individuals achieve serum 25(OH)D concentrations of 50 nmol/L or higher. Reanalysis suggests that 600 IU/day achieves levels above 26.8 nmol/L for only 97.5% of individuals. Higher doses needed to achieve target levels: To achieve the IOM target of 50 nmol/L for 97.5% of individuals, a significantly higher daily intake, potentially around 8895 IU, may be needed, significantly exceeding the current RDA and even the safe upper limit of intake. This underscores the scale of the current deficiency [27,28,29]. The results of our research are consistent with the presented analysis.

Beyond dose, co-factors and administration details may modify the biochemical response to D3 without guaranteeing changes in process metrics: co-supplementation with magnesium and vitamin K2, adequate fat co-ingestion, and sufficient intake of vitamin A, zinc, selenium, boron, as well as antioxidant status (e.g., glutathione, which can epigenetically modulate hepatic vitamin-D metabolism genes and VDR expression in obesity models) [11,12,13,29]. We did not collect standardized data on dose, duration, timing relative to extraction, or co-supplementation; accordingly, it is premature to draw the firm boundary that vitamin D has no effect on process outcomes. A pragmatic next step would be controlled prospective studies evaluating whether optimized vitamin D status prior to extraction influences manufacturing outcomes. Alternatively, controlled ex vivo evaluation of vitamin D metabolites in culture media may help clarify potential mechanistic effects under standardized process conditions.

The lack of an association between 25(OH)D and yield likely reflects the dominant influence of critical process parameters, which may override more subtle donor-related biological effects under standardized manufacturing conditions. Several factors may contribute to this observation, including the dominant influence of process parameters and the lack of standardized supplementation data.

Implications for GMP Translation and Manufacturing (autologous vs. “off-the-shelf”): The results emphasize that, for autologous ATMPs, process reproducibility is key: standardizing cutting (scissor cutting), selecting type II collagenase, and controlling the number of passages can meaningfully improve manufacturing efficiency and consistency, regardless of the fluctuations in recipients’ systemic status. In the context of off-the-shelf (allogeneic) products, our data strengthen the view that GMP-level refinement of CPPs and the use of qualified reagents are of primary importance for scalability and batch quality, whereas “patient optimization” at an insufficient level (e.g., D3 supplementation) may be of secondary importance for yield and process metrics per se. In particular, scissor-based preparation was associated with an approximately five-fold higher median yield compared with mechanical methods, and type II collagenase demonstrated a consistently superior performance across the yield, passages, and cryovial metrics, directly supporting their prioritization in standardized manufacturing.

Strengths and limitations: Strengths include: a large sample with complete 25(OH)D measurement; a prespecified analytical plan (nonparametric tests, BH-FDR, OLS with HC3); model diagnostics; and a log10(yield) sensitivity analysis with stable conclusions. Limitations: (i) observational design and lack of randomization with respect to D3 supplementation (risk of residual confounding); (ii) unequal group sizes in some enzyme levels (wider CIs; interpretive caution); (iii) no assessment of certain potentially relevant quality biomarkers (e.g., phenotype/surface markers, differentiation potential, OMICS); (iv) single-center setting and one SOP, which limits the generalizability to other laboratories; (v) incomplete control of time-to-processing and exact hormonal timing (morning vs. non-morning cortisol), which may modestly modulate outcomes; and (vi) absence of standardized data on the dose, duration, timing of D3 before extraction, and co-supplementation (K2, Mg, fat co-ingestion, A, Zn, Se, boron, and antioxidants), which limits inference on dose response and potential effect modification.

From the perspective of clinical implementation and GMP, we recommend:

− Standardizing preparation to scissor cutting and type II collagenase;
− Strict control of the number of passages (as a key CPP) and consideration of operational limits/thresholds;
− Maintaining consistent enzymatic–mechanical conditions, as these most strongly determine yield.

Future directions:

The present findings highlight the dominant role of critical process parameters in determining DPSC manufacturing efficiency. However, the potential contribution of donor-related biological factors, including vitamin D status, remains an open question requiring controlled investigation. Future prospective and mechanistic studies may evaluate whether the optimization of vitamin D status prior to tissue collection, or controlled ex vivo modulation of vitamin D signaling within culture systems, influences manufacturing outcomes when critical process parameters are rigorously standardized.

Such studies should incorporate clearly defined supplementation protocols, timing relative to tissue collection, and standardized manufacturing workflows to distinguish process-driven effects from donor-related biological variability. In addition, experimental approaches evaluating vitamin D metabolites directly within the culture environment may help clarify whether vitamin D signaling influences cellular proliferation, expansion efficiency, or other manufacturing-relevant characteristics.

These investigations may help to determine whether targeted biological optimization can provide incremental benefits beyond process standardization alone, while preserving the primary importance of critical process control in ensuring reproducible and scalable ATMP manufacturing.

## 4. Materials and Methods

### 4.1. Study Design and Ethics Approval

We conducted a prospective observational cohort study including a total of 250 consecutive adult patients who were referred for a planned extraction of impacted mandibular third molars for medical indications. Only healthy and well-formed teeth were included in the study [15,30]. Procedures were performed solely for clinical reasons, not for the purposes of the study.

After obtaining written informed consent from each participant, blood was drawn for laboratory assays (including 25(OH)D, PTH, Ca, inorganic phosphate, ALP, cortisol, GH, and complete blood count), and dental pulp from the extracted teeth was collected for cell isolation and culture according to the standard operating procedure (SOP) [30]. The study protocol received ethical approval from the Bioethics Committee at Wroclaw Medical University (approval no. MW34/2020).

The primary endpoints were serum 25(OH)D concentration and DPSC yield. Secondary endpoints included the number of passages and the number of cryovials obtained per sample.

To ensure clear alignment between clinical sampling, laboratory processing, and statistical analysis, all samples were processed according to the SOP using one defined preparation method (scissor cutting or mechanical dissociation) and one enzyme condition (e.g., type II collagenase, alternative enzyme, or no enzyme), reflecting the routine manufacturing practice. Samples were not split or processed using multiple methods in parallel. Based on these process parameters, samples were categorized into groups for a comparative analysis of manufacturing outcomes. In parallel, participants were stratified according to donor-related systemic factors, including vitamin D supplementation status and measured serum 25(OH)D levels. This structured framework enabled an integrated evaluation of both process-related and donor-related determinants of DPSC manufacturing efficiency.

### 4.2. Diagnostic and Demographic Parameters

As part of the laboratory evaluation, serum 25-hydroxyvitamin D (25(OH)D, ng/mL) was measured as an indicator of vitamin D status; cholecalciferol (D3) supplementation was documented in binary form (yes/no). In parallel, parathyroid hormone (PTH, intact, pg/mL), total calcium (Ca, mmol/L), and inorganic phosphate (P, mg/dL) were measured, as well as alkaline phosphatase activity (ALP, U/L). Cortisol (µg/dL) was assessed, with the time of blood draw recorded when a morning sample was available due to circadian variation. In addition, a growth hormone (growth hormone (GH), expressed in ng/mL according to the laboratory’s analytical procedure) and a complete blood count (e.g., WBC, RBC, Hb) were obtained. Demographic variables (age and sex) were obtained from the study’s database and analyzed descriptively, with age expressed as mean ± standard deviation and sex presented as counts and percentages.

### 4.3. Standard Operating Procedure (SOP) for Pulp Harvesting and Cell Isolation

**Figure 2:**
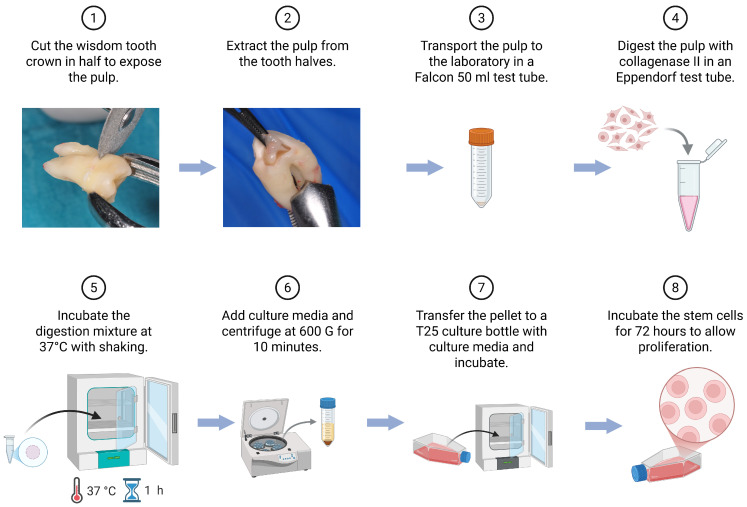
Isolation and primary culture of dental pulp stem cells (DPSCs). Schematic representation of the isolation procedure for human dental pulp stem cells. The tooth is cut into two halves to expose the pulp, which is gently removed and placed in a sterile tube for transport to the laboratory. The pulp tissue is then minced with sterile scissor cutting, digested in collagenase II, and incubated at 37 °C with shaking. After digestion, a culture medium is added, and the suspension is centrifuged at 600× *g* for 10 min. The resulting cell pellet is resuspended in the culture medium, transferred to a T25 flask, and incubated under standard conditions. After 72 h, adherent DPSC colonies become visible. Created in Biorender. Gębarowski, T. (2026). BioRender.com/025cvek. accessed in 2 March 2026.

**Figure 3:**
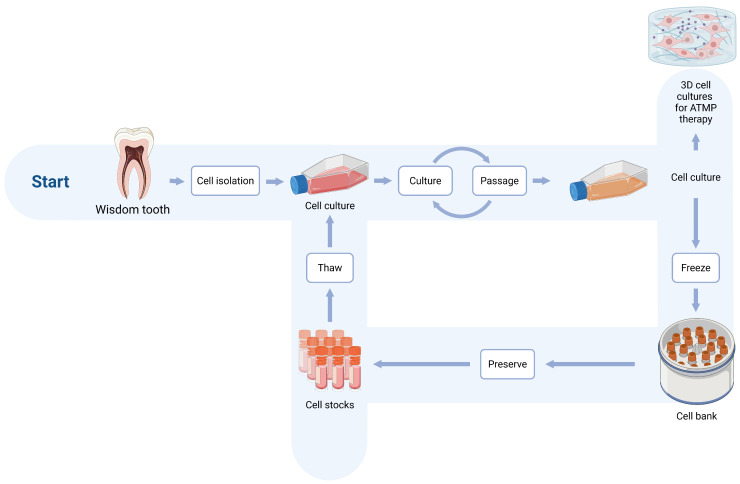
Process flow for dental pulp stem cells (DPSC) manufacturing under the study standard operating procedure (SOP): tooth extraction, pulp retrieval, mechanical fragmentation, optional enzymatic digestion, primary culture, passaging, cryopreservation and thaw, and (optionally) 3D culture for ATMP use. Created in BioRender. Gębarowski, T. (2026). BioRender.com/edkkmqb. accessed in 2 March 2026.

Procedures were analogous to our previously published work and were conducted under the same SOP (Figure 2) [30]. Immediately following tooth extraction under local anesthesia, the intact tooth was placed into a sterile antibiotic solution (gentamicin, 0.5 mg/mL; Sigma-Aldrich, St. Louis, MO, USA) maintained at 4 °C and transported to the laboratory under a cold chain. Under aseptic conditions in a laminar flow hood (BSL-2), the crown/root complex was sectioned ex vivo at the cemento-enamel junction using a diamond disk with continuous cooling, the pulp chamber was exposed, and the pulp tissue was gently retrieved with microsurgical forceps/excavators. The tissue was flushed with PBS containing gentamicin, then mechanically minced (scalpel/scissor cutting). Where indicated, fragments were enzymatically digested with collagenase II (4 mg/mL; Worthington Biochemical Corporation, Lakewood, NJ, USA, 37 °C, 60 min); in some preparations, no enzymes were used, relying on mechanical dissociation alone. Digestions were neutralized with a complete growth medium, optionally passed through a cell strainer as per SOP, and centrifuged at 200 g for 5 min at 4 °C. The pellet was resuspended in a pre-equilibrated growth medium and seeded onto cultureware; cells were maintained under standard incubation conditions and, after adhesion, expanded and passaged according to the SOP (Figure 2 and Figure 3) [19,31,32].

Quality control comprised sterility testing (including mycoplasma), assessment of viability and morphology, and, in selected samples, verification of minimal mesenchymal stromal cell criteria (plastic adherence; CD105/CD73/CD90 positivity with a lack of CD45/CD34/CD14 or CD11b/CD79a or CD19/HLA-DR; tri-lineage osteo-/adipo-/chondrogenic differentiation) [31]. All actions were documented in the SOP worksheet (time, operator, and deviations), and samples were coded (patient ID, tooth, and date/time) to preserve the audit trail [33]. All procedures were performed by trained personnel according to the standardized SOP, minimizing inter-operator variability.

### 4.4. Culture Conditions, Passaging, and Confluence

Primary seeding was performed in T25 flasks immediately after isolation. Cultures were maintained in a humidified incubator at 37 °C and 5% CO_2_ in α-MEM-based complete growth medium prepared according to the SOP and to prior work [1,30]. The media formulations were: (1) α-MEM (Thermo Fisher Scientific, Waltham, MA, USA) (with ribonucleosides/deoxyribonucleosides), FBS 10% (*v*/*v*; Gibco, Thermo Fisher Scientific, Waltham, MA, USA) (acceptable range: 10–20% depending on expansion needs), Penicillin–Streptomycin 1% (*v*/*v*) (final: 100 U/mL penicillin, 100 μg/mL streptomycin; Gibco, Thermo Fisher Scientific, Waltham, MA, USA), L-glutamine 2 mM (Gibco, Thermo Fisher Scientific, Waltham, MA, USA) and (2) MSC Nutristem XF Basal Medium with Supplement Mix (Biological Industries, Kibbutz Beit-Haemek, Israel) were changed every 48–72 h or earlier if pH/turbidity indicated the need to do so. The first check occurred at 72 h, then every subsequent check occurred at 48–72 h to assess attachment, morphology and contamination. Where sufficient cell numbers were available, seeding densities followed the SOP; in practice, 5 × 10^3^ to 1 × 10^4^ cells/cm^2^ were used during early expansion [33].

Confluence was judged by phase-contrast microscopy on the % of surface covered; growth dynamics (cell spreading, colony fusion, and mitotic activity) were documented at 48–72 h intervals. Passaging was performed at ≥80% confluence using TrypLE (typical exposure 2–5 min at 37 °C; Gibco, Thermo Fisher Scientific, Waltham, MA, USA), followed by neutralization with complete medium and reseeding at the target split ratio (1:2–1:4, per SOP). Subsequent passages proceeded in T75/T175 flasks under standard conditions (visual microscopic inspection) [33]. For experiments that were scheduled for osteogenic differentiation, confluence targets and timing matched the previously published protocol to ensure comparability [32]. All cultures were handled under aseptic conditions. Routine QC comprised sterility checks (including mycoplasma), viability and morphology.

### 4.5. Viability Assessment

Viability was assessed using AO/PI (Acridine Orange/Propidium Iodide) according to the manufacturer’s instructions; cell counts were obtained automatically (Countstar or equivalent device; Countstar, ALIT Life Science, Shanghai, China). Proliferation parameters (e.g., doubling time) were derived from serial readings/monitoring as in [34].

### 4.6. Thawing, Post-Thaw Viability Assessment, and Seeding of Cryopreserved Cells

Cryogenic vials of cells were stored in liquid nitrogen (≤−150 °C) in a complete medium containing 10% (*v*/*v*) DMSO (Sigma-Aldrich, St. Louis, MO, USA). For thawing, a single vial was removed rapidly from the cryobank and immediately placed in a 37 °C water bath; it was gently swirled until only a small ice crystal remained (typically ~60 s) to minimize exposure to DMSO at elevated temperature. The contents were aseptically transferred into a tube containing a pre-warmed (37 °C) complete medium at a 1:10 volume ratio, which was then centrifuged at 200× *g* for 5 min at room temperature. The DMSO-containing supernatant was removed due to dryness, and the pellet was gently resuspended in a fresh, warm complete medium; any small aggregates were dispersed by gentle trituration (slow, repeated pipetting) without using a sieve/filter. From this suspension, an aliquot was taken for a viability assessment by trypan blue exclusion: the cell suspension was mixed 1:1 with 0.4% trypan blue, incubated for 2–3 min at room temperature, and loaded by capillarity into a Bürker counting chamber (depth 0.100 mm, large-square area 1 mm^2^; volume per large square 1 × 10^−4^ mL). Unstained (transparent; viable) and blue-stained (non-viable) cells were counted separately, applying the standard boundary rule (include top and left borders; exclude bottom and right borders). Counting covered at least four large squares or ≥200 cells, then was repeated on a second loading. Cell concentration and viability were calculated from the mean counts as [21]:

$$Viability\left(\%\right)=V^{-}+D^{-}V^{-};\;C_{viable}=V^{-}\times 10^{4}\times DF;\;C_{total}=\left(V^{-}+D^{-}\right)\times 10^{4}\times DF$$

where V^−^ is the mean number of viable cells per large square; D^−^ is the mean number of non-viable cells per large square; 10^4^ derives from the volume of a large square (1 × 10^−4^ mL); and DF is the dilution factor of the sample with dye (DF = 2 for the 1:1 mixture used here). After viability determination, cells were seeded into culture vessels: a total number of cells up to 5 × 10^3^–1 × 10^4^ cells/cm^2^ were placed in T25 flasks or T75 flasks; alternatively, seeding was adjusted to a target surface density of >1 × 10^4^ cells/cm^2^. Flasks were pre-warmed, and cultures were maintained at 37 °C, 5% CO_2_, and >95% humidity. The medium was changed after 12–18 h to remove residual DMSO and weakly adherent cells, and it was exchanged for a fresh medium every 48–72 h. For study analyses, the “post-thaw viability” endpoint used the first measurement that was taken ≤30 min after thaw completion; the vial ID, thaw time, absolute and percentage viable cell counts, seeding density, and vessel ID were documented.

### 4.7. Statistical Analysis

Statistical analyses were performed in Python 3.11 (pandas, scipy, statsmodels, and matplotlib). Continuous variables were presented as mean ± SD when they were approximately normally distributed and were otherwise presented as median [IQR] and range. Categorical variables were presented as *n* (%), with 95% CIs (Wilson) reported for selected proportions. Normality was assessed with the Shapiro–Wilk test and inspection of plots; homogeneity of variance was inferred from residual plots. For two-group comparisons, Welch’s *t*-test was used when parametric assumptions were met; otherwise, the Mann–Whitney U test was applied. The effect sizes reported were Cohen’s d (with Hedges’ correction) or rank-biserial r. For comparisons of ≥3 groups, the Kruskal–Wallis test was used with Mann–Whitney post hoc tests and multiple comparison control via the Benjamini–Hochberg procedure (BH-FDR). Categorical variables were compared with the chi-square test or Fisher’s exact test when expected counts were small.

Bivariate associations were evaluated using Spearman’s correlation (primary), with BH-FDR control; for relationships with a clearly linear character (passages ↔ yield), Pearson’s correlation was additionally reported.

Endpoints: (1) 25(OH)D (ng/mL)—a marker of vitamin D status; (2) cell yield (million). Secondary analyses included the number of passages and the number of cryovials. In OLS models, robust HC3 standard errors were used. Main models for yield included systemic markers (25(OH)D, PTH, Ca, P, cortisol, and GH), demographic variables (age and sex), D3 supplementation, and process factors (isolation method and enzyme/medium); in an extended analysis, the number of passages was added. For 25(OH)D, determinants were examined with D3 supplementation (and—if available—the season was derived from the blood draw date).

To assess whether the effects of method/enzyme differed by supplementation status, models with interaction terms were fitted: supplementation × C (method) and supplementation × C (enzyme) (*p* values for interactions were reported). Collinearity was assessed using VIF (threshold 5). Model diagnostics were conducted graphically (residuals vs. fitted, QQ plot, Cook’s distance, and leverage). In sensitivity analysis, a log10(yield) transformation was applied in OLS; additional approaches (robust regression/Poisson) were considered and noted if they did not change conclusions. The two-sided tests were α = 0.05.

## 5. Conclusions

In this prospective observational cohort, critical process parameters (CPPs)—particularly tissue preparation using scissor cutting, enzymatic digestion with type II collagenase, and control of passage number—had a decisive impact on the manufacturing efficiency of dental pulp-derived stem cells. These factors were strongly associated with higher cell yield, expansion capacity, and cryovial production under the standardized SOP applied in this study.

In contrast, although vitamin D3 supplementation was associated with higher circulating 25(OH)D concentrations, neither vitamin D status nor supplementation was significantly associated with manufacturing outcomes, including cell yield, number of passages, or cryovial count. These findings indicate that the optimization and the strict control of CPPs represent the primary determinants of reproducible and efficient DPSC manufacturing.

From a translational and GMP perspective, prioritizing standardized preparation methods and enzymatic protocols may improve manufacturing consistency, scalability, and overall process robustness in ATMP workflows. Future controlled investigations may help determine whether donor vitamin D status optimization or ex vivo modulation of vitamin D signaling influences manufacturing outcomes under standardized process conditions.

## Figures and Tables

**Table 1:** Population characteristics by vitamin D3 supplementation. Abbreviations: ALP, alkaline phosphatase; PTH, parathyroid hormone; GH, growth hormone; DPSC, dental pulp stem cell; and 25(OH)D, 25-hydroxyvitamin D. Groups: no D3 supplementation (*n* = 161) vs. D3 supplementation (*n* = 89). Continuous variables are shown as mean ± SD when both groups were normally distributed (Shapiro–Wilk *p* ≥ 0.05), otherwise they are shown as median [IQR]. Between-group tests: Welch’s *t*-test or Mann–Whitney U, as appropriate. Categorical variables: chi-square/Fisher. Effect sizes: Cohen’s d or rank-biserial r for continuous and Cramér’s V for categorical.

Variable	No D3 Supplementation	D3 Supplementation	Test	*p*	Effect Size
Age (years)	24.0 [5.0]	27.0 [10.0]	Mann–Whitney U	0.0558	rank-biserial r = −0.16
Sex: Female (n, %)	110 (68.3%)	74 (83.1%)	Chi-square	0.0166	Cramér V = 0.15
25(OH)D (ng/mL)	23.3 [13.2]	34.2 [20.5]	Mann–Whitney U	0.0000	rank-biserial r = −0.52
PTH (intact) (pg/mL)	30.8 [15.5]	30.7 [14.0]	Mann–Whitney U	0.6342	rank-biserial r = −0.04
Calcium (Ca) (mmol/L)	2.37 ± 0.08	2.38 ± 0.10	Welch *t*-test	0.3833	Cohen d = 0.15
Phosphates (P) (mg/dL)	3.22 ± 0.57	3.37 ± 0.66	Welch *t*-test	0.1083	Cohen d = 0.25
Alkaline phosphatase (ALP) (U/L)	61.0 [25.5]	61.0 [22.5]	Mann–Whitney U	0.2952	rank-biserial r = 0.09
Cortisol (µg/dL)	10.8 [7.8]	11.4 [8.4]	Mann–Whitney U	0.9058	rank-biserial r = 0.01
Growth hormone (GH) (ng/mL)	0.7 [3.1]	1.2 [3.4]	Mann–Whitney U	0.5466	rank-biserial r = −0.05
Cell yield (million)	4.0 [4.2]	5.0 [4.0]	Mann–Whitney U	0.1184	rank-biserial r = −0.15
Passages (count)	2.0 [1.0]	2.0 [0.0]	Mann–Whitney U	0.0956	rank-biserial r = −0.13
Cryovials (count)	3.0 [5.0]	5.0 [4.0]	Mann–Whitney U	0.1598	rank-biserial r = −0.20
25(OH)D status: Deficit (<20) [n (%)]	50 (36.0%)	8 (10.1%)	Chi-square	0.0001	Cramér V = 0.24
25(OH)D status: Insufficiency (20–29.9) [n (%)]	48 (34.5%)	19 (24.1%)	Chi-square	0.1943	Cramér V = 0.08
25(OH)D status: Optimal (≥30) [n (%)]	41 (29.5%)	52 (65.8%)	Chi-square	0.0000	Cramér V = 0.32

Footnotes: Units: Ca—mmol/L; P—mg/dL; ALP—U/L; PTH—pg/mL; 25(OH)D—ng/mL; cortisol—µg/dL; GH—ng/mL; and yield—million cells. The 25(OH)D status definition is as follows: deficiency < 20 ng/mL; insufficiency 20–29.9 ng/mL; and optimal ≥ 30 ng/mL. Presentation: mean ± SD when both groups were normal (Shapiro–Wilk *p* ≥ 0.05), otherwise median [IQR]. Tests: Welch’s *t*-test or Mann–Whitney U (continuous) and chi-square/Fisher (categorical). Effect sizes: Cohen’s d/rank-biserial r/Cramér’s V.

**Table 2:** Key associations and multivariable estimates. Values are median [IQR] unless stated otherwise. Correlations by Spearman with BH–FDR control; two-group contrasts by Mann–Whitney; and multivariable estimates by OLS with HC3 robust SEs. NS = non-significant.

Outcome	Predictor	Metric	Estimate (95% CI)	*p*/p(FDR)	Note
Yield (million)	Passages	Pearson r	0.69	<10^−26^	Spearman ρ 0.82, *p* < 10^−44^
Yield (million)	25(OH)D	Spearman ρ	0.14	0.168	NS after FDR
Passages (n)	25(OH)D	Spearman ρ	0.07	0.406	NS
Yield (million)	Passages	β (OLS-HC3)	2.28 (1.86–2.69)	<10^−26^	per 1 passage
Yield (million)	25(OH)D	β (OLS-HC3)	0.02 (−0.01–0.05)	0.30	per 1 ng/mL
25(OH)D (ng/mL)	D3 supplementation	β (OLS-HC3)	+13.2 (7.4–19.0)	8.1 × 10^−6^	R^2^ ≈ 0.29

**Table 3:** Preparation method (scissor cutting vs. mechanical). The values are median [IQR]. *p* from Mann–Whitney U. Effect size = rank biserial *r*.

Outcome	Scissor Cutting	Mechanical Tearing/Pipetting	*p*-Value	Effect Size
Cell yield (million)	5.00 [3.00]	1.00 [0.50]	3.6 × 10^−13^	r = 0.82
Passages (n)	2.00 [0.00]	0.00 [0.25]	1.5 × 10^−17^	r = 0.81
Cryovials (n)	5.00 [5.00]	1.00 [0.00]	7.4 × 10^−4^	r = 0.62

Footnote: higher medians and large r values consistently favor scissor cutting.

**Table 4:** Enzyme/medium, group medians and global tests. The values are median [IQR]. Global comparison by Kruskal–Wallis; BH-FDR post hoc Mann–Whitney: significant pairs remained for yield (3), passages (6), and cryovials (10).

Outcome	Type II Collagenase	Collagenase/Dispase	None/Low Dose	Kruskal–Wallis *p*
Cell yield (million)	5.00 [3.00]	≈1.00	≈1.00	6.8 × 10^−15^
Passages (n)	2.00 [0.00]	0.00–1.00	0.00–1.00	6.2 × 10^−22^
Cryovials (n)	5.00 [4.00]	0.00–1.00	0.00–1.00	8.4 × 10^−8^

Note: The largest post hoc effects favored type II collagenase vs. dispase/none (e.g., yield r up to 0.93, p(FDR) < 0.05).

## Data Availability

The original contributions presented in this study are included in the article. Further inquiries can be directed to the corresponding author.
